# Motivations for Voluntary Participation in the Food Sanitation and Safety Management System, Patterns of Non-Conformance Reports, and Recertification Improvement Trends Among Food Businesses in Taiwan

**DOI:** 10.3390/foods14213784

**Published:** 2025-11-04

**Authors:** Shin-Yu Chen, Keng-Wen Lien

**Affiliations:** 1 Department of Food Science, National Pingtung University of Science and Technology, Pingtung 912301, Taiwan; sychen@mail.npust.edu.tw; 2 Continuing Education Program of Food Biotechnology Applications, College of Science and Engineering, National Taitung University, Taitung 95092, Taiwan

**Keywords:** food safety, food sanitation and safety management system certification, second-tier quality control

## Abstract

This study investigates the motivations behind food businesses voluntary participation in certification programs and analyzes their non-conformance reports (NCRs). Health and nutrition food businesses lead in voluntary certification, averaging 5.65 NCRs per business, with common non-conformances primarily related to miscalibrated instruments. Meat processing businesses follow closely, with their main non-conformance being inadequate maintenance of environmental cleanliness. The primary motivation for seeking certification among these food businesses is driven by the requirements of international trade and export markets. Furthermore, from 2016 to 2023, businesses undergoing recertification demonstrated a significant reduction in NCRs, decreasing from an average of 11.17 to 5.17. Certification not only enhances food hygiene and safety management but also facilitates the efficient acquisition of export health certificates from the Taiwan Food and Drug Administration. Therefore, we encourage all food businesses to engage in certification programs to elevate their hygiene and safety standards and thereby contribute to food safety assurance.

## 1. Introduction

In the current global food supply chain, food safety is a critical issue that affects everyone, from producers to consumers [1]. Millions of people worldwide fall ill annually from consuming contaminated food, particularly in developing nations, making food safety a pressing societal challenge [2]. To ensure a healthy and secure food supply, international organizations have established various Food Safety Management Systems (FSMS). These systems help food businesses identify, assess, and control food safety hazards [1].

The Hazard Analysis and Critical Control Point (HACCP) system is an internationally recognized tool used to pinpoint potential problems in food production and determine preventive measures. Good Manufacturing Practices (GMP) are considered Prerequisite Programs (PRPs) for HACCP, designed to establish a hygienic environment for safe food production. GMP emphasizes the control of the entire production environment, equipment, personnel hygiene, and process control [1]. Furthermore, the International Organization for Standardization (ISO) has issued auditable international standards, such as ISO 22000:2018 [3], which secure food safety across the entire supply chain and provide a globally harmonized framework [3,4,5]. These management systems are not just regulatory requirements but are often key to accessing international markets. For instance, ISO 22000:2018 emphasizes Risk-Based Thinking and the PDCA (Plan-Do-Check-Act) cycle for achieving continuous improvement and compliance [3,4,5]. Effective implementation of these standards has been shown to significantly reduce production downtime and Non-Conformance Reports (NCRs), thus enhancing operational efficiency and product quality.

Beyond mandatory regulations, many companies pursue voluntary certifications, such as BRC-GS (Brand Reputation through Compliance Global Standards), IFS (International Featured Standard), ISO 22000 [3,4,5,6], and those required for cultural or religious needs, like Halal Certification [1]. The adoption of these voluntary standards is typically driven by several managerial motivations: (1) Commercial Motivation: Many companies implement FSMS to increase sales, gain a competitive advantage, and access international markets that mandate these standards. For example, in emerging economies like those in Latin America, commercial motives are a primary factor driving effective FSMS implementation as companies seek internationalization [6]. (2) Ethical Motivation: This aims to uphold corporate principles and ensure consumer welfare. In regions with higher food safety awareness, such as Europe, ethical motives are considered a significant driver for effective FSMS implementation [6]. (3) Legitimacy Motivation: This focuses on meeting legal requirements and customer expectations [6]. However, research indicates that the mere pursuit of legitimacy (superficial implementation just to meet social norms) may negatively impact the effective implementation of FSMS, particularly in less regulated environments [6].

The implementation challenges and effectiveness of FSMS vary by industry and company size. The food manufacturing sector is dominated by Small and Medium-sized Enterprises (SMEs), which constitute the majority of businesses in many national economies (>90%). Yet, SMEs often face resource constraints that hinder their ability to comply with food safety requirements, resulting in lower compliance rates [1]. NCRs types also differ across sectors. Studies on multi-line facilities (e.g., dairy, meat processing, and bakery) found that implementing ISO 22000:2018 can significantly reduce non-compliance incidents, demonstrating the standard’s effectiveness in complex production settings [1]. The root causes behind these NCRs are usually multifaceted [1,2,3,4,5,6]: (1) Insufficient Knowledge and Awareness: Employees lack basic food hygiene knowledge, or management lacks commitment to a food safety culture, leading to poor personal hygiene or neglected documentation. A significant inverse correlation between training hours and performance improvement indicates that increased training can effectively improve operational outcomes. (2) Financial Constraints: SMEs often lack the capital to invest in GMP-compliant facilities, equipment calibration, cleaning programs, or employee health checks. (3) Inadequate Management Support and Supervision. (4) A lack of commitment from senior management and insufficient on-site supervision can lead to resource shortages and increase employee resistance to change.

Taiwan’s Act Governing Food Safety and Sanitation imposes strict accreditation standards on Certification Bodies (CBs) and penalizes food businesses officially notified by the government for failing to obtain the mandatory FSSMS certification or Second-Tier Quality Control (STQC) [7]. These legislative improvements aim to strengthen the overall food safety regulatory framework. Currently, over 500 food enterprises are mandated to obtain FSSMS certification. Despite the mandatory requirements for certain sectors, some food businesses outside the compulsory categories voluntarily participate in the certification program. While existing literature confirms the general benefits of FSMS, there remains a gap about the specific motivations for voluntary participation in the mandatory certification system within Taiwan’s context.

This study aims to identify the motivations driving voluntary participation in the FFSSMS among Taiwanese food businesses and to evaluate the benefits of voluntary certification by analyzing NCRs and related outcomes. Using certification data from firms that completed at least two FSSMS certification cycles between 2016 and 2023, the study will (1) characterize firms’ stated reasons for continued engagement, (2) quantify changes in NCRs patterns across certification cycles, and (3) assess how firm attributes (e.g., industry, capital size, and region) are associated with certification outcomes, thereby providing evidence to inform managerial practice and policy support for autonomous food-safety management.

## 2. Materials and Methods

### 2.1. Certification of FSSMS

#### 2.1.1. Certification Regulations

Pursuant to Article 8, Paragraphs 5 and 6 of the Act Governing Food Safety and Sanitation, the following regulations have been established [8]: (1) Accreditation of CBs and sanitation and safety control of food businesses of certification regulations. (2) Certification operation procedures for FSSMS CBs. (3) Certification operation procedures for FSSMS. (4) Charging measures for certification and accreditation of FSSMS.

#### 2.1.2. Scope of Certification

The Certification of FSSMS is systemic for factory premises, not product-based [8]. It adheres to the standards of Good Hygiene Practice (GHP) as per Article 8, Paragraph 1, and the HACCP system as per Paragraph 2 of the Food Safety Act. However, only food enterprises of specific categories and scales designated by the TFDA are mandated to comply with the HACCP system.

#### 2.1.3. Certification Bodies (CBs)

The certification of FSSMS is conducted by organizations accredited by the TFDA, including food Industry Research and Development Institute, China Grain Products Research and Development Institute, National Animal Industry Foundation, and Association for the Development and Certification of Agricultural Products. These CBs must comply with the accreditation of CBs and sanitation and safety control of food businesses of certification regulations, possess an ISO 22003 certification [9], appoint full-time auditors, and provide relevant education and training for the auditors.

#### 2.1.4. Certification Process

(1) Online application: Food businesses must log into the Food Safety Accreditation and Certification System (FACS) using a commercial certificate or an authorized personal certificate to submit their application. The system will then perform a conflict of interest screening and randomly assign cases to strengthen the fairness and independence of the certification process [10].

(2) Document review: After the system randomly assigns a case, the applicant must submit the relevant certification documents to the CB for a document review. If there are NCRs during the review period, the food businesses must submit an improvement report as per regulations.

(3) On-site audit: Before the on-site audit, the CB will confirm the evaluation plan with the food businesses, including the date, auditors, and process. Auditors may request that any production line within the scope of the application be operational during the on-site audit. If NCRs are found, the food businesses must submit a report detailing the improvements made in accordance with the regulations.

(4) Certification deliberation: CB will deliberate and determine the outcome of the certification. If approved, a certificate of certification will be issued to the food businesses [8]. If the certification is not approved, the food businesses will be given the opportunity to present their case before being notified of the final result.

### 2.2. Sampling Strategy

This study used the Taiwan Food and Drug Administration’s Food Safety Accreditation and Certification System (FACS) as the data source. We conducted a census of firms that met the following inclusion criteria: (1) voluntarily participated in FSSMS certification; (2) completed at least two full certification cycles between 2016 and 2023; and (3) were not subject to government-mandated certification. These inclusion criteria were applied using FACS records to ensure the selected sample reflects the practice of voluntary, repeated certification. After screening, 52 food businesses met the inclusion criteria and were included in the subsequent data processing and analysis.

### 2.3. Certification Result Analysis

Using the FACS database (https://facs.fda.gov.tw) accessed on 10 January 2024, food businesses that voluntarily completed two full certification cycles of the FSSMS program between 2016 and 2023, and are not subject to mandatory certification requirements, were selected for analysis. Their certification reports were reviewed to examine their motivations for application, capital size, industry distribution, regional allocation across counties and cities, as well as common NCRs and certification pass rates.

Regarding capital size classification, based on the Food Manufacturer Management Planning Blueprint issued by the Taiwan Food and Drug Administration (TFDA) under the Ministry of Health and Welfare [11], food manufacturers are categorized according to their factory registration status and capital investment. The classification thresholds are defined as below NTD 30 million, between NTD 30 million and NTD 100 million, and above NTD 100 million [10].

### 2.4. Hypotheses

The objectives of this study are as follows: (1) To identify the managerial motivations driving firms’ continued, voluntary participation in FSMS certification. This exploration aims to uncover the fundamental reasons for autonomous FSMS implementation [6]. (2) To quantify changes in NCRs patterns across successive certification cycles. This is hypothesized based on the core FSMS principle of continuous improvement [4]. (3) To assess how firm attributes (e.g., industry, capital size, and region) are associated with certification outcomes. This investigation is motivated by prior literature, which found that GMP non-conformities among Malaysian SMEs—such as inadequate facility design, missing documentation, and poor operational control—reflect significant industry and structural constraints [1].

Based on the above research objectives, this study hypothesizes the following: (1) H1: The number of NCRs observed at recertification will be significantly lower than at initial certification. This hypothesis reflects the expectation that firms reduce non-conformances and residual risks through continuous improvement and verification processes. (2) H2: The number of NCRs differs across industries. This hypothesis is based on the premise that industry characteristics and operating conditions shape hazard profiles and management difficulty, so different food sectors will show systematic differences in the frequency and types of non-conformances. (3) H3: Firm capitalization (size) influences the number of NCRs. The rationale is that a firm’s size affects resource availability, internal governance, and its capacity to respond to external pressures, which can in turn affect food safety compliance performance.

### 2.5. Statistical Analysis

Data collected from the certification reports were analyzed using SPSS 17 statistical software. A one-way analysis of variance (ANOVA) was employed to examine differences in mean values, and descriptive analysis was conducted. The results were presented in the form of frequency and percentage (%) distributions. The analysis included the correlation between food industry categories, capital amounts, and the occurrence of NCRs.

### 2.6. Qualitative Interviews

To understand witness evaluators’ on-site observations and perspectives regarding firms that voluntarily participate in FSSMS certification, we used purposive sampling and invited three TFDA witness evaluators who have long participated in and witnessed FSSMS audits to take part in in-depth interviews. The interviews were intended to capture the evaluators’ practical observations of voluntary participants during audits.

Participants provided informed consent before the interviews. Written consent was obtained prior to each interview. Interviews were semi-structured and primarily open-ended, allowing respondents to freely describe their observations and judgments during on-site audits; interviewers asked follow-up questions as needed to clarify details. Interviews were conducted in the participants’ preferred language (Chinese) and lasted approximately 30–45 min each. With participants’ permission, interviews were audiorecorded, transcribed verbatim, and used for subsequent analysis. For the qualitative data analysis, the verbatim transcripts were processed and systematically examined using Thematic Analysis. This study did not involve interventional human trials, and given the nature of the data and interviews, no additional clinical ethics review was required. Interview guide (main questions) as follow:

(1) Based on your observations, does a firm’s continued participation in certification lead to changes in its food safety management? What are the possible reasons?
(2) Based on your observations, do the number or patterns of NCRs differ across industries? What are the possible reasons?
(3) Based on your observations, does a firm’s capital size affect the number of NCRs or the speed of improvement? What are the possible reasons?
(4) Based on your observations, why do food businesses voluntarily participate in certification?

## 3. Results and Discussion

### 3.1. Certification Pass Rate

By the end of 2023, a total of 52 food businesses had voluntarily completed two full cycles of the FSSMS certification program, achieving a 100% pass rate. This success rate is notably higher than the approximate 90% pass rate observed among food businesses mandated to undergo certification [10]. Observations from witness evaluations suggest that most voluntarily certified food businesses demonstrated greater confidence in their food hygiene and safety management systems. They also conducted thorough preparations prior to the certification process, which likely contributed to their full compliance and successful outcomes.

Research limitations, the sample in this study is highly selective, potentially introducing a positive selection bias:

(1) Voluntary Participation and Survivor Bias: Firms included in this study had to meet strict criteria: voluntary (non-mandatory) FSSMS participation and completion of at least two full certification cycles (2016–2023). This resulted in a small final sample of only 52 firms.

(2) High-Motivation and High-Performance Sample: These firms—which voluntarily participated and successfully achieved recertification—typically demonstrated superior preparation and a 100% pass rate. Compared to the approximately 90% pass rate for mandatory certification firms, the voluntary sample represents a group with a significantly higher level of food safety commitment and internal governance structure”.

### 3.2. Capital of Food Businesses Voluntarily Applying for FSSMS Certification

According to the TFDA’s Food Manufacturer Management Planning Blueprint [11], food enterprises are categorized into three capital thresholds: below NTD 30 million, between NTD 30 million and NTD 100 million, and above NTD 100 million [10]. Among voluntarily certified food businesses, those with capital investments below NTD 30 million account for 19.23%, companies with capital between NTD 30 million and NTD 100 million represent 9.62%, and those with capital exceeding NTD 100 million constitute the majority at 71.15%. This distribution indicates that food businesses with higher capital investment levels are more inclined to participate voluntarily in the FSSMS certification program.

**Figure 1:**
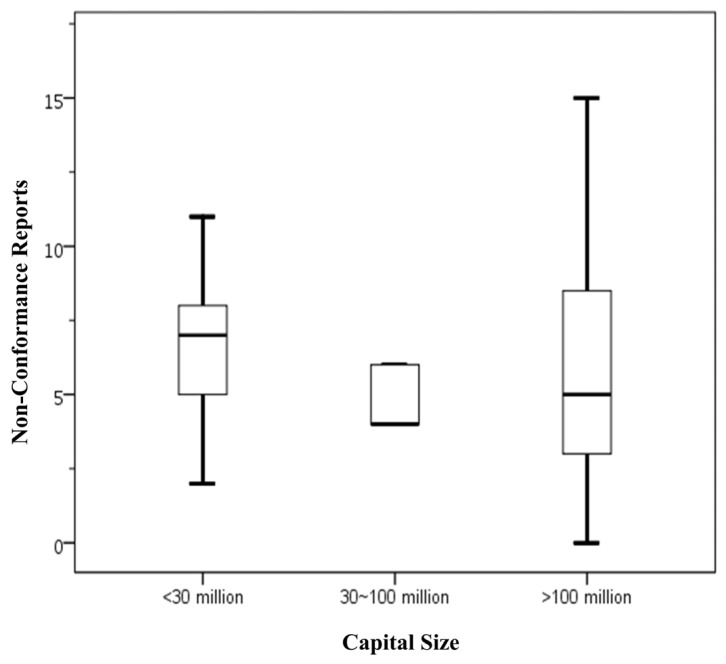
Number of NCRs by capital size in food businesses voluntarily applying for FSSMS certification program. Means sharing the same letter are not significantly different at the 5% significance level based on the Least Significant Difference test. Box plots display data from bottom to top as follows: minimum, first quartile, median, third quartile, and maximum.

Figure 1 presents statistical data on the number of NCRs issued to food businesses with varying capital investments that voluntarily applied for FSSMS certification. The findings are as follows: food businesses with capital below NTD 30 million had an average of 6.50 NCRs (Standard deviation (SD) = 2.68), with a range from 2 to 11 NCRs. Those with capital between NTD 30 million and 100 million showed a lower average of 4.80 NCRs (SD = 1.10), ranging from 4 to 6 NCRs. Food businesses with capital exceeding NTD 100 million exhibited an average of 6.41 NCRs, but with a considerably wider variation (SD = 4.70), spanning from 0 to 20 NCRs. Overall, the total sample of 52 businesses had an average NCRs count of 6.27 (SD = 4.15), with a range from 0 to 20. The data indicate that businesses with the largest capital investments demonstrated the greatest variability in NCRs numbers, although their mean NCRs was comparable to that of the smallest capital group. Conversely, businesses with mid-level capital investments had the lowest average NCRs count, but this difference was not statistically significant. Given the limited sample size, further data collection is needed to validate these trends.

In conclusion, the current analysis suggests that capital investment does not have a significant impact on the number of NCRs. This finding is consistent with previous studies involving mandatory certification participants [10], indicating that capital size is not directly associated with the quality of food safety management within these businesses.

Hasnan et al. summarized common NCR categories as inadequate sanitary design and facilities, weak documentation, poor cleaning and maintenance, and deficiencies in operations control and training [1]. Macheka et al. in a study of Zimbabwe’s food sector, traced such non-conformities to structural constraints including financial shortages, infrastructure deficits, limited top management commitment, and insufficient technical support [12]. These two studies help explain why documentation/calibration deficiencies and sanitary-facility NCRs persist in smaller plants; they also account for why structured certification—with its systematization and training requirements—often yields substantial NCRs reductions at recertification, since the certification process can correct the institutional and capability gaps described above. Although these deficiencies are frequently linked to financial constraints, our data show that large firms with greater capital still present the same types of NCRs, indicating that lack of funding is not the sole or primary determinant of NCRs occurrence; organizational governance, management commitment, institutionalized practices, and staff capability are also key influencing factors.

Research limitations, Capital and Industry Concentration Bias. The characteristics of the final sample exhibit significant skewness regarding firm attributes. Specifically, the sample is dominated by large enterprises, with 71.15% reporting capital exceeding NTD 100 million. Furthermore, the industry representation is highly concentrated in the health and nutrition food manufacturing and meat processing sectors, most of which possess an export-oriented motivation. Consequently, the conclusions of this study primarily reflect the behavioral patterns of firms that are resource-sufficient, actively seeking a competitive advantage, and are subject to international market pressures. This intrinsic characteristic limits the external generalizability of the findings when applied to SMEs or firms under mandatory certification. SMEs, which often face financial shortages and infrastructure constraints, may exhibit different NCRs patterns and have varying capacities for achieving continuous improvement compared to the enterprises observed in this study.

### 3.3. Geographic Distribution of Food Businesses Voluntarily Applying for FSSMS Certification

Table 1 summarizes the voluntary participation in FSSMS certification by food businesses across different regions and counties/cities in Taiwan.

In Northern Region: Constitutes 40% of the total sample with 21 businesses. Within this region, New Taipei City accounts for 6 businesses (12% of the total), Taoyuan City leads with 11 businesses (21%), while Hsinchu County and Yilan County each host 2 businesses, representing 4%, respectively.

In Central Region: Makes up 19% of the total with 10 businesses. Taichung City comprises 5 businesses (10%), and Changhua County, Nantou County, and Yunlin County have 1, 1, and 3 businesses, respectively, corresponding to 2%, 2%, and 6% of the total.

In Southern Region: Also represents 40% of the total with 21 businesses. Tainan City has the highest concentration with 15 businesses (29%), followed by Kaohsiung City with 3 businesses (6%). Chiayi City, Chiayi County, and Pingtung County each host 1 business, each contributing 2%.

This distribution reflects a pronounced clustering of food businesses in the Northern and Southern regions of Taiwan. Notably, the Northern region’s concentration is centered in New Taipei City and Taoyuan City, while the Southern region is dominated by Tainan City. These three municipalities—Tainan, Taoyuan, and New Taipei City—account for the highest proportions of food businesses voluntarily seeking FSSMS certification.

The prominence of these locations is largely attributable to their well-developed industrial and business parks supporting the food manufacturing sector. According to the Ministry of Economic Affairs factory registry [13], New Taipei City hosts 1319 registered food manufacturing businesses, followed by Tainan City with 800, and Taoyuan City with 753 establishments. The high density of food manufacturers in these municipalities likely drives the greater voluntary certification participation, as businesses seek to enhance their competitive advantage through formal food safety and sanitation management systems.

Research limitations, Sample Size and Representativeness. A significant limitation of this study is the highly constrained sample size and its representativeness. The overall sample includes only 52 firms that voluntarily completed two full certification cycles. This count is highly limited compared to the total number of food manufacturers in Taiwan (e.g., 1319 registered food manufacturers in New Taipei City alone). Consequently, the findings must be interpreted cautiously, as the results primarily reflect a small, high-performing subgroup of the industry, which inevitably affects the external generalizability of our conclusions.

### 3.4. Industry Proportion of Food Businesses Voluntarily Applying for FSSMS Certification

Among the food businesses voluntarily applying for FSSMS certification, the Health and Nutrition Food Manufacturing sector leads with a 19% participation rate, highlighting a demand for certification within this industry. The Meat Processing sector follows, accounting for 10% of voluntary certifications, ranking second in industry engagement. The remaining categories, collectively grouped as Other Food Products, represent the majority with 71% of voluntary certification participation. This broad category encompasses diverse industries, including Seasoning Manufacturing, Flour Milling Products, Edible Ice Products, Food Utensils, Containers and Packaging, Noodle and Vermicelli Manufacturing, Seafood Processing, Non-Alcoholic Beverage Manufacturing, Baking and Steaming Food Manufacturing, Vegetable and Fruit Processing, Agricultural Product Processing, Chocolate and Candy Manufacturing, Ready-to-Eat Meals, Egg Products, Tea Manufacturing, Starch and Starch Product Manufacturing, Meal and Dish Manufacturing, among others.

**Figure 2:**
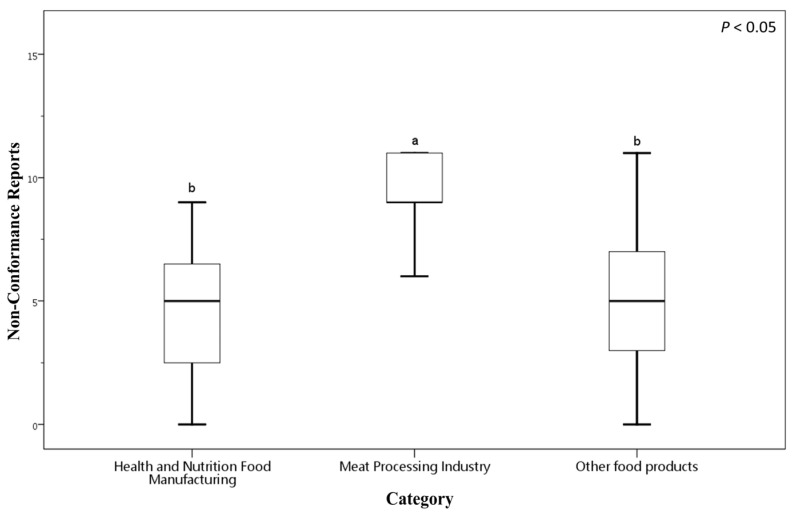
Number of NCRs among food businesses of different categories voluntarily applying for FSSMS certification. Different letters (a, b) indicate significant differences among the mean NCR values at the 5% level based on Duncan’s multiple range test. Box plots display data from bottom to top as follows: minimum, first quartile, median, third quartile, and maximum.

Figure 2 presents a box plot illustrating the distribution of NCRs across these food manufacturing categories. The Health and Nutrition Food Manufacturing sector exhibits a mean NCRs of 5.65 (SD = 4.45), indicating variability in compliances. In Health and Nutrition Food Manufacturing sector, NCRs values range from 0, where some factories had no NCRs, to a maximum of 15. The Meat Processing sector shows a notably higher mean NCRs of 10.56 (SD = 4.59), with NCRs counts ranging from a minimum of 5 to a maximum of 20 (the highest recorded in the certification reports). The Other Food Products category reports a mean NCRs of 5.86 (SD = 4.50), comparable to the Health and Nutrition sector, with NCRs values spanning from 0 to 20.

These data indicate that the Meat Processing Industry faces greater challenges in meeting FSSMS certification requirements, as reflected by significantly higher average NCRs counts and wider variability. In contrast, the Health and Nutrition Food Manufacturing and Other Food Products sectors tend to perform better, reporting lower average NCRs. Nonetheless, substantial variation within these groups suggests heterogeneous levels of food safety compliance among individual businesses.

### 3.5. Motivations for Food Businesses to Apply for FSSMS Certification

Among food businesses voluntarily participating in the FSSMS certification, health and nutrition product manufacturers represent 19%, while meat processing manufacturers account for 10%. According to certification reports, these higher participation rates are primarily driven by international trade and export requirements. For example, health supplements exported to Malaysia must obtain FSSMS certification before applying for the GMP certificate issued by the TFDA, which serves as a mandatory document for exports to Malaysia. Similarly, exports to other Association of Southeast Asian Nations countries such as Vietnam and Indonesia also require GMP certification [14].

In addition, certain international buyers demand that meat processing manufacturers hold recognized food safety certifications. As a result, domestic meat processors pursue FSSMS certification to demonstrate regulatory compliance and build trust with foreign clients. Beyond export considerations, many food businesses voluntarily seek FSSMS certification to enhance consumer confidence and convey their commitment to food safety. Other voluntarily certified food businesses indicate that their primary motivation is to improve market competitiveness, followed by fulfilling requirements from domestic distributors.

Drivers identified in China for implementing food safety certification: state ownership, media attention, mission statement salience, and firm size, operating in multiple effective configurations, suggest that external visibility and internal normative commitments can catalyze certification [15]. However, these drivers were not observed in Taiwan food businesses. Rincon-Ballesteros et al. examine the implementation of FSMS in agri-food firms across different regional contexts and find that the institutional environment significantly moderates motivations for adoption: ethical considerations are a primary driver in Europe, whereas commercial motivations are more influential in Latin America and legitimacy seeking motives may exert a negative effect [6]. In Taiwan, voluntary participation in certification programs appears to be mainly performance and market oriented, resembling the motivational profile observed in Latin America.

### 3.6. NCRs in GHP Among Food Businesses Voluntarily Applying for FSSMS Certification

Table 2 outlines the common NCRs where food businesses, voluntarily applying for FSSMS certification, have reported non-conformances with GHP. The data, based on a sample size of 104, encompasses both initial certification and recertification phases, the results are as follows:

(1) Calibration of Instruments: The most frequent NCRs, at 22%, involve measuring instruments like thermometers, pressure gauges, and scales not being regularly calibrated or their calibration range not covering the commonly used measurement range.
(2) Equipment Compliance: 19% of NCRs indicate that equipment, utensils, and containers used in food production do not meet the required operational, usage, and maintenance standards for hygiene and safety.
(3) Facility Cleanliness: Accounted for 16% of NCRs, the deficiency in maintaining clean floors and ceilings to prevent issues such as mold, peeling, and other hygiene-related concerns is noted.
(4) Pest Control: Also representing 16% of NCRs, there is documented evidence of pest activity within the facility, indicating a need for more rigorous pest management protocols.
(5) Foreign Object Management: Comprising another 16%, there is a noted lack of effective measures in place to prevent the contamination of food products with foreign materials during the manufacturing process.
(6) Traceability of Raw Materials: 15% of NCRs are related to the use of raw materials without adequate traceability information or records.
(7) Acceptance of Raw Materials: 11% of NCRs are due to raw materials not undergoing proper acceptance procedures upon receipt.
(8) Inspection Status Management: Another 11% of NCRs are due to improper marking and management of the inspection status of raw materials, semi-finished, and finished products.
(9) Management of Chemicals: 10% of NCRs are associated with cleaning agents, disinfectants, and toxic chemicals not being managed and recorded by a designated individual.
(10) Process Control: Also at 10%, there are reports of lacking established control methods and standards for critical process parameters such as temperature, humidity, pH, water activity, pressure, flow rate, or time.

**Table 2:** Common GHP NCRs among food businesses voluntarily applying for FSSMS certification.

Rank	Number of NCRs of GHP	Percentage (%) *n* = 104 ^a^
1	Thermometers, pressure gauges, scales, and other measuring instruments are not regularly calibrated, or the calibration range does not cover the commonly used measurement range.	22%
2	Equipment, utensils, and containers used in the food production process do not comply with operational, usage, and maintenance standards for hygiene and safety.	19%
3	Floors or ceilings are not kept clean to prevent mold growth, peeling, dust accumulation, grime, or condensation.	16%
4	Evidence of pest infestation in the facility area.	16%
5	Effective measures are not taken to prevent the contamination of food with metal or other foreign objects during the production process.	16%
6	The raw materials used lack traceability information or records.	15%
7	Raw materials are not subjected to a proper acceptance procedure upon receipt.	11%
8	The inspection status of raw materials, semi-finished, and finished products in the process is not appropriately marked and managed.	11%
9	Cleaning agents, disinfectants, and toxic chemicals are not managed and recorded by a designated person.	10%
10	For food processes that require control of temperature, humidity, pH, water activity, pressure, flow rate, or time, related control methods and standards have not been established, nor have records been maintained.	10%

^a^ Initial certification and recertification of 52 food businesses.

The data reveals that the most common challenges faced by food businesses relate to the calibration of measuring instruments and compliance with equipment standards. These are critical for ensuring product safety and quality, suggesting a need for enhanced focus on regular maintenance and verification protocols. The tied issues at 16% reflect a concern for overall facility hygiene and product integrity, emphasizing the importance of comprehensive sanitation and preventive controls. Traceability and proper acceptance of raw materials are essential for supply chain transparency and food safety, indicating that more rigorous procedures may be necessary. The management of inspection statuses and chemicals points to a need for better documentation and control practices. Lastly, the control of process parameters is fundamental to consistent quality, food businesses could benefit from improved monitoring systems and record-keeping. These analysis underscores the importance of addressing these common NCRs to not only meet certification standards but also to uphold the integrity of the food safety management system and ensure consumer trust. It is crucial for food businesses to implement corrective actions and continuous improvement strategies to mitigate these issues. In the study conducted by Halim et al. [16], the prevalence of NCRs within halal-certified small to medium-sized enterprises in Malaysia was examined, alongside the determinants of such incidents. The research pinpointed several pivotal factors that contribute to the occurrence of NCRs, including a deficiency in employee awareness, inadequate commitment from upper management, and a scarcity of staff allocated for documentation tasks, compounded by misunderstandings pertaining to regulatory guidelines. In this study, the NCRs improvement responses indicated that the most common root cause analysis of NCRs was inadequate employee education and training, and to strengthen and ameliorate the NCRs, educational training methods were employed. Insufficient training of employees can result in a lack of awareness among employees and misconceptions regarding regulatory guidelines.

Among food businesses participating in voluntary FSSMS certification from 2016 to 2023, those in the Health and Nutrition Food Manufacturing sector are the most numerous. The primary NCRs for these businesses is the lack of regular calibration of measuring instruments such as thermometers, pressure gauges, and scales. The second most common issue is that the calibration range of these instruments does not cover the measurement ranges commonly used. The third most frequent NCRs involves the equipment, utensils, and containers used in the food production process, which do not comply with the principles of hygiene and safety in their operation, use, and maintenance, along with evidence of pest infestation in the facility area. Due to regulatory requirements [14], health and nutrition food manufacturers must label the packaging of their products with health or nutritional claim indicators, including the components and their quantities. If the measuring instruments are inaccurate, it will severely impact the accuracy of these indicator components’ quantity labels on the product packaging. Hasnan et al. examined 20 scholarly articles published between 2012 and 2021 that addressed GMP in the context of small-scale food processing operations [1]. The review highlighted that inadequate sanitary design and facilities were the most frequently reported issues, mentioned in 85% of the articles. In the current study, the Health and Nutrition Food Manufacturing sector similarly reported inadequate sanitary design and facilities as the most frequently encountered issue.

Meat processing businesses are the second most numerous in voluntary participation in Food Safety Certification. Their primary deficiency is the failure to maintain cleanliness of floors and ceilings, leading to issues such as mold growth, peeling, dust accumulation, grime, or condensation. The second most common NCRs is the lack of regular food safety, hygiene, and quality management training for on-duty staff, along with a failure to document such training. The third concern is the use of raw materials without traceable source information or records. An unclean environment can lead to microbial contamination or the introduction of foreign objects into meat products. Additionally, the absence of traceable source information complicates the investigation of issues when they arise. For example, if there are residues of veterinary drugs in the product, it is essential to trace the origin of the meat to determine the cause of the problem. The study by Jakubowska-Gawlik et al. observed substandard levels of hygiene in meat processing sector and their casing suppliers, with poor storage and foreign matter control measures [17]. Similarly to the NCRs of meat processing businesses trend observed in this study.

The GHP standards encompass the management regulations for the operation sites, facilities, and quality assurance systems used by food businesses in the manufacturing, processing, mixing, packaging, transporting, storing, and selling of food or food additives. These standards are designed to ensure the hygiene, safety, and quality of food products. An analysis of the top ten deficiencies in GHP among food businesses that voluntarily apply for FSSMS certification revealed that issues with measuring instruments are the most common. This NCRs is similar to those found in businesses undergoing mandatory certification [10], indicating a lack of calibration and judgment concepts for measuring instruments among Taiwan food businesses. There is a deficiency in systematic management of instruments within the factory premises, suggesting a need for strengthened guidance and educational training. Whether it is valuable instruments and advanced processing equipment or simple measuring instruments used daily, if regular calibration and maintenance are not performed, at best, insufficient freezing temperatures can cause raw materials to spoil and be wasted; at worst, it can lead to direct or indirect failure of process control, endangering product quality and safety, and resulting in significant reputational damage.

Other common NCRs include the equipment, utensils, and containers used in the food production process, where their operation, use, and maintenance do not comply with hygiene and safety principles and fail to maintain environmental cleanliness in the factory area. For example, there may be traces of vectors in the factory, or floors and ceilings are not kept clean to prevent mold growth, peeling, dust accumulation, fouling, or condensation. Additionally, entrances, doors, windows, ventilation ports, and other openings are not kept clean. Furthermore, accurate records are not kept or preserved, such as the use of chemicals, traceability of raw materials, or gaps in education and training records. These deficiencies are also commonly found in businesses undergoing mandatory certification [10]. Food businesses may enhance educational training regarding the aforementioned deficiencies to prevent the recurrence of these issues.

Regarding the limitations of this study, a potential source of variability stems from the CBs involved. The professional expertise and experience of different CBs are not uniform. For example, the National Animal Industry Foundation possesses a long-standing specialization in meat product certification, which may grant it an inherent advantage when auditing meat-related food safety standards. Furthermore, the professional competence and experience levels of individual auditors employed by different CBs can vary, potentially leading to inconsistencies in audit findings and the number of NCRs issued.

Second, the scope of the FSSMS certification presents a key limitation. FSSMS certification primarily focuses on the technical dimensions of food safety, such as GHP and HACCP systems. The audit scope is typically confined to hygiene and safety related elements, and it does not explicitly assess softer management components, including food safety culture, leadership, or management commitment. Consequently, this study was unable to investigate the influence of these crucial factors on the effectiveness of plant hygiene and safety management, nor could it assess their role in driving continuous improvement.

Finally, the reliance on certification reports and NCRs as the primary data source is a notable limitation. The data analysis is susceptible to the quality of documentation and potential differences in reporting standards across various audits. Although the reduction in NCRs over successive certification cycles suggests improved management effectiveness, the study lacks in-depth qualitative data to explore the underlying reasons and specific management mechanisms responsible for these improvements. Therefore, future research is recommended to incorporate qualitative methods, such as interviews with both management and employees, to gain a more nuanced under-standing of how food safety culture and leadership commitment influence safety performance within a food processing facility.

### 3.7. Statistical Result of NCRs for Initial Certification and Recertification

**Figure 3:**
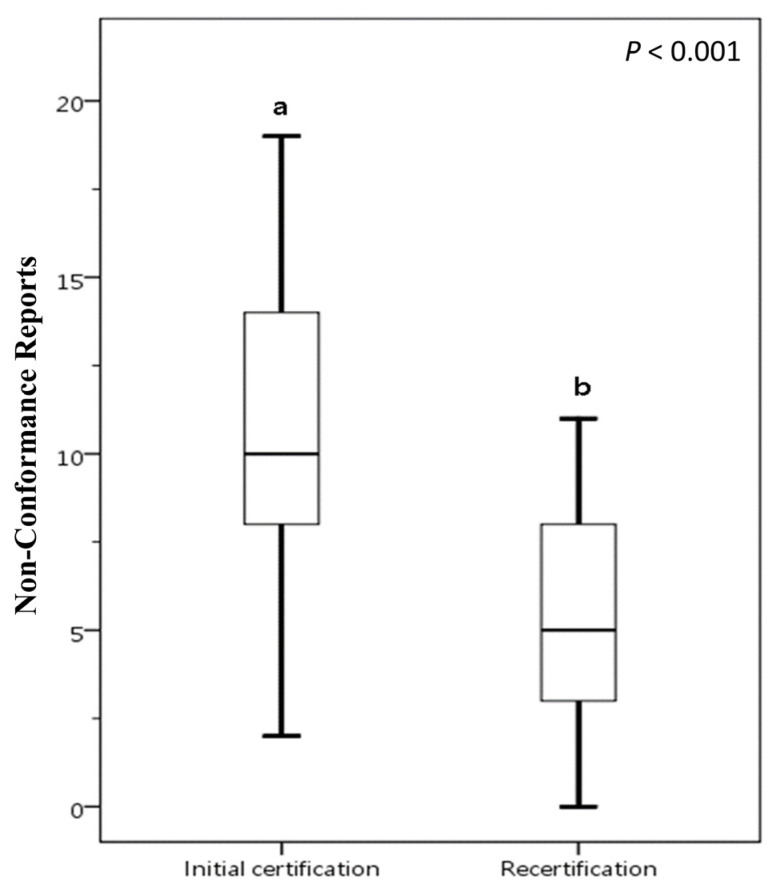
Comparative analysis of NCRs between initial certification and recertification for food businesses voluntarily participating in FSSMS certification program. The presence of different letters (a, b) indicates a statistically significant difference in the mean NCR values (*p* < 0.05). Box plots display data from bottom to top as: minimum, first quartile, median, third quartile, and maximum.

A comparative analysis was conducted on food businesses that underwent voluntary participation FSSMS certification twice between 2016 and 2023, as illustrated in Figure 3. In the initial certification, the mean number of NCRs is 11.17, with a SD of 5.82. This indicates that, on average, there are just over 11 NCRs during the initial certification process. In the recertification, the mean number of NCRs drops to 5.17, with a SD of 3.13. This suggests that there are fewer issues during recertification, and the lower SD points to less variability compared to the initial certification. The data suggests that the initial certification process presents more challenges for food businesses. In contrast, the recertification process tends to have fewer NCRs. This improvement may be attributed to the valuable experience acquired during initial certification or to enhancements implemented FSSMS subsequently. The results demonstrate that certification through the FSSMS indeed aids food businesses in strengthening or maintaining the quality of their safety and sanitation management. This also indicates that participating in FSSMS certification helps achieve the goal of ensuring access to safe, nutritious, and sufficient food for all people year-round.

Conceptually, the FSMS improvement pattern aligns with the ISO 22000:2018 framework process orientation, PDCA applied at both management and operational levels, and explicit risk-based thinking. Continued participation in certification operationalizes preventive controls and supports continuous improvement, thereby reducing the incidence of NCRs. Rihawi assessed the impact of implementing the ISO 22000:2018 Food Safety Management System on the performance of food facilities with multiple production lines by comparing key performance indicators before and after implementation [5]; the study reported measurable improvements in operational efficiency, product safety, and compliance. However, the author noted that the short-term nature of the study limits insight into the long-term sustainability of these improvements. Our study confirms that long-term implementation of an FSMS can also produce significant improvements in factory food safety compliance. Taken together, these findings suggest both immediate measurable gains and the potential for sustained improvement under prolonged FSMS implementation.

Research limitations in NCRs Data Interpretation. The interpretation of the observed significant reduction in NCRs across certification cycles (from a mean of 11.17 to 5.17) must be contextualized by the inherent limitations stemming from selection bias and data dependency. (1) Selection Bias and Overestimation of Improvement. The sample used in this study is highly susceptible to positive selection bias (or survivor bias). Since the sample exclusively comprises firms that voluntarily participated in FSSMS and successfully completed at least two full certification cycles, it inherently excludes companies that dropped out or failed recertification. This suggests that the significant improvement trend observed may be overestimated, as it primarily reflects the superior managerial commitment and endogenous capabilities of these high-performing firms, rather than an improvement effect universally applicable to all participating enterprises. Data Dependency and Misclassification Risk. Our analysis relies entirely on audit reports and NCRs records, making the results vulnerable to factors outside of actual production performance, primarily due to misclassification bias and data subjectivity:

- Subjectivity in Audit Standards: Despite standardized procedures for certification, the professional competence and experience of individual auditors can vary, leading to inconsistent standards in judging “non-conformities” under GHP/HACCP requirements. This subjectivity may result in similar underlying defects being recorded as different quantities or types of NCRs across distinct audits, or introduce classification bias (e.g., miscategorizing an issue as instrument calibration rather than process control).
- Discrepancy in Reporting: The observed reduction in NCRs numbers during recertification might be partly attributable to firms learning to “manage the audit” or improving their documentation quality, rather than realizing substantial, physical improvements in production processes. This disparity between documentation and reality could distort the classification of the firm’s actual performance.

### 3.8. Qualitative Findings from Interviews with TFDA Witness Evaluators

The interviewees reported that they seldom have the opportunity to witness audits of the same company in close succession, which makes systematic comparisons of initial certification versus recertification at the individual firm level difficult. However, they observed that firms voluntarily pursuing certification generally undertake more thorough preparation prior to audits. Interviewees also indicated that third-party audits help identify weaknesses and that firms tend to institutionalize training and internal audit mechanisms; these measures can effectively reduce the frequency and severity of common non-conformances such as instrument calibration, facility cleanliness, foreign object control, and raw material traceability. Consistent with these observations, the quantitative data show a significant reduction in mean NCRs from 11.17 at initial certification to 5.17 at recertification, a result that may be related to the mechanisms described above.

The interviewees consistently observed differences between industries. They attributed these differences to factors such as the age of facilities and variations in hygienic design, as well as inherent industry risks. They noted that meat processing plants are often housed in older facilities with aging equipment and instruments, which are more difficult to maintain. Constraints related to equipment and workflow, combined with entrenched work practices and a resistance to changing documentation culture, make environmental sanitation and record keeping more prone to NCRs.

Interviewees generally did not observe a stable, direct association between capital size and the number of NCRs. Some small firms performed well, whereas large firms often exhibited greater variability in NCR counts. Large firms are more able to invest in equipment replacement, routine calibration, environmental maintenance, dedicated QA personnel, structured training programs, internal audits, and digital monitoring; these investments tend to increase institutional maturity and operational stability. However, insufficient management commitment or organizational complexity can also produce high variability in performance and NCRs outcomes. Small firms whose owners prioritize food safety and implement rigorous SOPs and traceability procedures can likewise achieve low NCRs counts. In summary, capital is a facilitating condition but not a sufficient one; management commitment and governance structures are equally critical for reducing NCRs and sustaining food safety performance.

The interviewees identified the main drivers of firms’ voluntary certification as international trade and export requirements (e.g., obtaining GMP or sanitary certificates for export), enhancing consumer trust, strengthening market competitiveness, and complying with domestic channel or buyer requirements. These external market and buyer pressures encourage firms to establish or improve GHP/HACCP systems earlier, which in turn leads to better audit performance and faster improvement cycles, thereby forming a positive feedback loop of continuous improvement.

The qualitative observations of the witness evaluators corroborate the study’s quantitative findings: NCRs at recertification decreased substantially, and industry sector and management maturity—rather than capital size alone—were the primary factors influencing NCRs patterns and improvement outcomes. Qualitative insights complemented the quantitative data by revealing causal mechanisms not directly observable in the quantitative analysis (e.g., how training, internal audits, and external market pressure drive improvement) and can serve as a basis for future research focusing on management commitment and organizational culture.

Regarding the limitations of this study, the qualitative component comprised in-depth interviews with three witness evaluators. Their perspectives provide professional depth but do not represent the views of all witness evaluators or audit personnel.

Research limitations, Depth and Representativeness of Qualitative Data. A significant limitation exists regarding the depth and breadth of the qualitative data. Although in-depth interviews were conducted with TFDA witness evaluators, their perspectives may not be fully representative of all auditing personnel or the overall industry experience. Critically, the study lacks extensive qualitative data from the firms themselves (e.g., interviews with management, quality assurance staff, and employees). Consequently, while the assessors’ insights provide crucial external validation and mechanism explanation for the observed NCRs reductions, the study cannot fully explore the internal management mechanisms and specific organizational processes that contributed to the sustained performance improvements from the companies’ perspective.

### 3.9. Benefits of Voluntary Application for FSSMS Certification

Based on audit reports and observations from witness evaluations, the benefits of voluntary application for FSSMS certification are summarized as follows:

(1) Enhancement of food safety and sanitation management: although food businesses are well-prepared before applying for the FSSMS certification, the process still uncovers some NCRs. Audits by third-party entities on GHP and HACCP within the food factory can help businesses identify and rectify deficiencies early on, allowing them to implement more robust management of food safety and sanitation and establish a more comprehensive control system for the food production process.
(2) Simplification of health certificate application process: for health certificate applications, records from the past six months showing compliance with GHP, as verified by health authorities, are recognized. Therefore, businesses that pass the FSSMS certification can reduce the application time for exporting food health certificates from 39 working days to 8 working days, and the cost from NTD 7300 to 3000 [18]. Additionally, no on-site inspections are required within the validity period, significantly shortening processing times and saving on-site inspection costs.
(3) Expansion of international trade markets: with the increasing frequency of international trade, many foreign buyers require businesses to pass food safety standard certifications. If businesses can pass FSSMS certification, it demonstrates that their factory production processes meet hygiene standards, aiding in the expansion into international markets.
(4) Winning Consumer Favor: As the demand for food safety among Taiwan consumers increases, food businesses that meet and pass the TFDA FSSMS certification standards can be verified by consumers through the TFDA FACS website. This transparency boosts consumer trust in food businesses and distinguishes them in the marketplace.
(5) Achieving the United Nations Sustainable Development Goals (SDGs): Obtaining FSSMS certification indeed helps food businesses enhance or maintain the quality of their safety and sanitation management, ensuring their product safety. For health and nutrition businesses, it ensures that the nutrient content of their products matches the labeling. These efforts contribute significantly to achieving the goal of SDG 2.1, which is to ensure access to safe, nutritious, and sufficient food for all people year-round.

### 3.10. Key Findings: Continuous Improvement and Industry Heterogeneity

1. Continuous Compliance and Improvement of FSSMS:

Our study’s findings confirm a significant reduction in the number of NCRs observed during recertification audits compared to the initial verification cycle (*p*-value < 0.001). This result provides strong empirical support for the value proposition of FSMS, confirming the theoretical framework of continuous improvement via the PDCA cycle. This finding is highly consistent with international research on standards such as ISO 22000 and BRCGS [3,4,5], corroborating a positive correlation between system operational maturity and compliance. Notably, the results obtained within Taiwan’s voluntary FSSMS certification framework also confirm this universal principle, providing new empirical support for food safety policy implementation in Taiwan.

Qualitative interview results offer a key mechanistic explanation for the reduction in NCRs. Witness evaluations consistently noted that the most significant improvements were achieved by companies whose top management not only allocated resources but also demonstrated active commitment and fostered a robust food safety culture. This transformation converted external verification requirements into a proactive internal risk management strategy, moving beyond mere reactive auditing. Conversely, firms exhibiting less significant improvement often incurred NCRs due to issues directly traceable to poor management oversight, such as inadequate employee training and intermittent record loss.

2. Heterogeneous Effects Across Industries:

Another key finding is the significant heterogeneity in both the quantity and type of NCRs across different industry categories (Health and Nutrition Food vs. meat processing). The sectoral difference in NCRs patterns is not merely a reflection of varying management capabilities, but rather an embodiment of industry-specific inherent risks identified during audits. NCRs in the meat processing industry concentrate on sanitation and cross-contamination control, reflecting the inherent challenges of high-humidity, high-bioburden environments. This aligns with international literature on risk assessments for the meat and dairy industries [17,19]. In contrast, NCRs in Health and Nutrition Food Manufacturing primarily concern instrument calibration or document labeling, highlighting the higher requirements for GMP rigor and regulatory compliance for product functional claims.

Qualitative analysis further demonstrated that SMEs with limited capital often face a critical misalignment in resource allocation. Assessors observed that SMEs struggle to simultaneously invest in infrastructure improvements (to address hygienic design deficiencies) and the implementation of complex quality assurance systems (e.g., robust document control). This fragmented resource allocation hinders the fundamental elimination of NCRs, which supports the literature arguing that financial constraints in SMEs negatively impact sustained regulatory compliance [16].

Therefore, this study argues that in promoting FSSMS, policymakers and CBs must transition from a unified standardization perspective to an industry-specific risk management approach, providing differentiated guidance and resource support to truly enhance the overall food supply chain safety.

## 4. Conclusions

### 4.1. Key Findings and Academic Contributions

This study provides strong empirical support for the benefits of voluntary food safety certification through quantitative analysis. We confirmed a sustained and significant reduction in the number of NCRs across certification cycles (from an average of 11.17 to 5.17 at recertification). This tangibly validates the effectiveness of the core principle of continuous improvement within FSMS, as embodied by the PDCA cycle and risk-based thinking under frameworks like ISO 22000:2018, over long-term implementation (2016–2023 data). This finding complements existing literature, which often focuses solely on the short-term benefits of FSMS implementation.

Furthermore, our analysis confirmed the heterogeneous nature of FSMS implementation outcomes: the quantity and patterns of NCRs are primarily influenced by industry category and Organizational Maturity, rather than merely capital size. We identified organizational governance, proactive management commitment, and institutionalized practices as critical mediating factors for FSMS success. Regarding motivation, Taiwanese firms’ voluntary participation is predominantly driven by performance and market orientation, aligning with similar commercial motivations observed in other geographical areas like Latin America, thereby enriching the empirical literature on how Institutional Theory influences corporate food safety decisions across diverse economic environments.

### 4.2. Implications and Recommendations

Recommendations for Industry Operators:

(1) Operational Priorities and Process Optimization: Firms should concentrate resources on the most frequent NCR categories, including establishing systematic calibration and maintenance plans for measuring instruments (particularly for the health food sector due to strict labeling requirements), strengthening environmental hygiene and pest control (critical for inherent challenges in the meat processing industry), and formalizing raw material traceability and acceptance procedures.
(2) Management Commitment and Human Resources: Commitment from owners and top management is the core driver for maintaining low NCRs. Large firms should ensure that management commitment effectively translates into daily institutionalized practices and regular staff training, rather than relying solely on financial advantage.
(3) Market Benefits of Certification: Firms should actively leverage the substantial benefits of FSMS certification, such as the simplification of export health certificate application procedures, to gain efficiency and competitive advantage in international trade and market access.

Recommendations for Administrative Bodies:

(1) Enhancing Incentives for Voluntary Participation: Policymakers should continue and expand support, such as administrative simplification and cost savings, for firms that achieve FSSMS certification to promote broader voluntary participation.
(2) Targeted Technical Assistance: Policy should provide tailored technical assistance and subsidized training, specifically targeting:
  - SMEs: To help overcome financial constraints and establish fundamental hygiene management and documentation systems.
  - High-Risk or Challenged Industries: To offer subsidies for infrastructure upgrading or systematic maintenance (e.g., for aging facilities in the meat processing sector) to address structural limitations.
  - Resolution of Common Deficiencies: To strengthen national guidance and training on pervasive issues like “instrument calibration”.
(3) Promoting SDGs: FSSMS certification should be utilized as a key strategy to advance the United Nations SDG 2.1, ensuring access to safe and nutritious food for all.

Recommendations for CBs: Given the potential for inconsistent audit outcomes due to differences in CBs and individual auditor interpretation, CBs should strengthen standardized training for auditors. Specifically, they must ensure consistent interpretation criteria for GHP/HACCP standards when making and classifying NCR judgments.

In conclusion, this study provides robust empirical evidence that voluntary FSSMS certification yields measurable and sustained NCRs reductions, delivering practical advantages in market entry and consumer trust. These findings offer actionable guidance for industry players, policymakers, and CBs seeking to enhance the effectiveness and reach of voluntary food safety certification.

## Figures and Tables

**Table 1:** Geographical distribution of food businesses voluntarily certified for FSSMS in Taiwan, by region and county/city.

Region	Number of Businesses	Percentage (%)	County/City	Number of Businesses	Percentage (%)
North	21	40%	New Taipei City	6	12%
			Taoyuan City	11	21%
			Hsinchu County	2	4%
			Yilan County	2	4%
Central	10	19%	Taichung City	5	10%
			Changhua County	1	2%
			Nantou County	1	2%
			Yunlin County	3	6%
South	21	40%	Tainan City	15	29%
			Kaohsiung City	3	6%
			Chiayi City	1	2%
			Chiayi County	1	2%
			Pingtung County	1	2%
East	0	0%	Hualien County,	0	0%
			Taitung County	0	0%
Total	52	100%	Taiwan	52	100%

## Data Availability

The original contributions presented in the study are included in the article, further inquiries can be directed to the corresponding author.

## Abbreviations

The following abbreviations are used in this manuscript:

ANOVA	Analysis of Variance
CBs	Certification Bodies
FACS	Food Safety Accreditation and Certification System
FSMS	Food Safety Management System
FSSMS	Food Sanitation and Safety Management System
GHP	Good Hygiene Practice
GMP	Good Manufacturing Practice
HACCP	Hazard Analysis and Critical Control Point
ISO	International Organization for Standardization
NCRs	Non-Conformance Reports
PDCA	Plan–Do–Check–Act
PRPs	Prerequisite Programs
SD	Standard Deviation
SDGs	Sustainable Development Goals
SMEs	Small and Medium-sized Enterprises
STQC	Second-Tier Quality Control
TFDA	Taiwan Food and Drug Administration
